# Supplementary Catalogue of Plants

**Published:** 1836

**Authors:** John L. Riddell

**Affiliations:** Adjunct Professor of Chemistry, and Lecturer on Botany, in the Cincinnati Medical College


### Art. III.—Supplementary Catalogue of Ohio Plants.

*Embracing the species discovered within the state of Ohio, in* 1835.* *Catalogue and descriptions read, and specimens exhibited, before the Western Academy of Natural Sciences, March* 16, 1836. By John L. Riddell, M. D., Adjunct Professor of Chemistry, and Lecturer on Botany, in the Cincinnati Medical College.

*Araliaceæ.*

Aralia hispida, *Michx.* Wild Elder. Dwarf elder. It frequents rocky woods, and grows about 18 inches high. In July, it is said to bear greenish white flowers. On the pine-clad, rubblestone knob, between Kirtland and Painesville, near the Mountain House. Berries nearly black,—ripe near the last of August. The decoction of the root is a powerful diuretic, and has acquired great fame in curing dropsy. It colors the hair black. Whitney's Family Physician, (1833,) p. 187. Dr. Donovan, late Prof. of the Practice of Medicine in Willoughby University, regards it as a good alterant.

*Umbelliferæ.*

Thaspium barbinode, *Nutt.* i. 196. Thapsia trifoliata, *Spreng.* Brushy prairie, Dayton. Stone's hill, Cincinnati. Flowers pale yellow with us,—in June.

*Ranunculaceæ.*

Ranunculus acris, *Linn.* Crowfoot. Butter-cup. Flowers yellow,—in May. Perennial, 1 to 2 feet high. Alum creek, Franklin co. *Paddock.* Acrid, rubefacient. Wood and Bache, 543.

* For a list of the vegetable productions, previously known to be indigenous to Ohio, the reader is referred to a Synopsis of the Flora of the Western States, published in 1835; or, to the Western Journal of the Medical and Physical Sciences, for January and April, of the same year.

R. aquatilis, *De Cand.* Inhabiting stagnant pools; stem 1 to 2 feet long, flowers white, appearing in July. Miami country, and central Ohio.

Coptis trifolia, *Salis.* Gold thread. Swamps in the Western Reserve.

*Papaveraceæ.*

Meconopsis petiolata, *Nutt.* Abundant on the rich, shaded hill-sides, near the Ohio river. Cincinnati, etc.

*Hydropeltideæ.*

Hydropeltis purpurea, *Michx.* Water target. Pools, Dresden and Akron.

Flœrkea uliginosa, *Muhl.* False mermaid. Dry hill, in open woods, Cincinnati. Worthington. Grows 8 or 10 inches high.

*Cruciferæ.*

Nasturtium natans, *De Cand.* Swimming water cress. Flowers pale yellow, appearing in July. Stem two feet long, submersed leaves pectinately divided. Stagnant pools, Bank lick, Ky. *T. G. Lea, Esq.*, Coshocton.

Arabis lyrata, *Linn.* A very delicate species, less than a foot high, growing in tufts among sandstone rocks, in Kentucky, opposite the mouth of the Scioto. Ross co. O.

Thlaspi campestre, *Linn.* River banks, etc. Cincinnati. Worthington.

*Grossulaceæ.*

Ribes floridum, *Willd.* Wild black currant. Akron.

R. lacustre, *Pursh.* Flowers small, greenish yellow, appearing in May. Three to four feet high, woody. Berries dark brown, hispid. Columbus. *Lapham.*

R. cynos-bati, *Jacq.* Prickly gooseberry. Dayton. *Mr. M. G. Williams.*

*Onagrariæ.*

Epilobium coloratum, *Muhl.* Rivulet beds, wet meadows etc. Marietta. Worthington. Dayton.

*Halorageæ.*

Proserpinaca pectinata, *Lam.* Mermaid weed. An annual aquatic, with finely divided leaves. Stem a foot long. Flowers green, appear in July. Pools, Dayton.

Myriophyllum ambiguum, *Nutt.* Stems 1 to 2 feet long. Leaves incrusted with calcareous (?) matter. Pools, two miles north of Newark, Licking co.

M. scabratum, *Michx.* Coneaught Lake, near Meadville, Penn., a few miles east of the Ohio line. *R. Buchanan.* Summit lake, Akron, O., where I have procured it of two yards length. It is noted by Elliott, and others, as being 1 foot long. Leaves all pinnatifid, segments capillary; flowers purple, in delicate, interrupted spikes.

M. spicatum, *Linn.* Water milfoil. Branching, 18 inches high. Flowers in August and September. Pond, near Mill creek bridge, Cincinnati. The M. verticillatum has not yet been discovered within the state.

Callitriche verna, var. intermedia, *Wild.* Water chickweed. In water running slowly through a swamp, Webbsport, Muskingum county.

*Salicariæ.*

Ammannia humilis, *Ell.* i. 218. Six inches high, flowers in July. Margin of a pond, 5 miles east of Cincinnati.

*Rosaceæ.*

Comarum palustre, *Willd.* Marsh five finger. Boggy situations in the Western Reserve.

Stylypus vernus, *Raf.* Med. Fl. i. 223. Grows abundantly in fields, meadows, and woods. Height 1 to 2 feet, flowers yellow, appearing in May, carpels in a globose head, stipitate so as to be raised out of the calyx. Has the habit of a Geum. Central and southern parts of Ohio, Kentucky etc.

Rubus saxatilis, var. canadensis, *Michx.* Rock bramble. Brier herb. Flowers white, appearing in June or July; fruit black. Western Reserve. Rocky hill, Chilicothe.

R. trivialis, var. flaggellaris, *Willd.* ? Procumbent, closely armed with slender, declined prickles, not hispid; *leaves* ternate, leafets glabrous, round-obovate, irregularly dentate-serrate. Bogs, Akron, among Sphagnums and Andromedas.

Rosa gemella, *Willd.* Perennial, 8 to 18 inches high; flowers large, numerous, pale red, appear in June. Dry, rocky situations. Dupuy's knob, Ky., opposite Portsmouth, O. Hills about Chilicothe.

R. lucida, *Willd.* 4 feet high. Wet. Worthington. June.

R. carolina, *Linn.* Columbus. *Lapham.*

R. ——? branching, 8 to 16 inches high, *prickles* straight, declined, larger ones often in pairs, smaller ones frequently crowned with a gland, or a tuft of stellated pubescence; *leaves* mostly in 5's, oval and obovate, obtuse, acutely serrate and subciliate, smooth above, sparingly pilose beneath; *stipules* lance-linear entire, acute, ciliate and tomentose; *petioles* tomentose; *peduncles* nearly glabrous; *germ* ovate, with a few pedicelled glands; *sepals* lanceolate, attenuate, with two or three linear branches, tomentose within, glandular hispid without, equalling the petals; *petals* large, 1½ inch long, by 1 inch in width, obovate, cuneate, retuse, nearly obcordate, pale red, damask red or almost white; *styles* free. Hills near Chilicothe. Highest part of Dupuy's sandstone knob, Ky., opposite the mouth of the Scioto river. Resembles what I have taken to be R. gemella, *Willd.*, with which it grows, and of which it may be a variety. Flowers in June.

Spiræa tomentosa, *Linn.* Hard-hack. A little shrub, 2 to 3 feet high, with purple flowers—in July and August. Damp meadows. Akron.

Gillenia trifoliata, *Mœnch.* Indian physic. Near Dayton. *Buchanan.*

*Pomaceæ.*

Pyrus angustifolia, *Ait.* Flowers in May. Franklin co. *Paddock.*

P. melanocarpa, *Willd.* A shrub, 5 to 7 feet high, colonizing marshes and quaking bogs, in the Western Reserve. Flowers in May and June. Fruit deep brownish red, almost black, equalling small peas in size, scarcely edible.

*Amygdaleæ.*

Prunus borealis, *Pursh*. Red choke cherry. A shrub, 6 to 12 feet high, flowering in May, and growing in woods in the Western Reserve. Fruit ripe in August, equalling small peas in size, astringent, but otherwise agreeable.

*Leguminosæ.*

Baptisia tinctoria, *Linn*. Wild indigo. Perennial, 2 to 3 feet high, flowers yellow—in July and August. Barrens, near Cleveland.

Lespedeza reticulata, *Pers*. Flowers violet, stem often branching, branches erect, leaves linear, oblong, mucronate. Flowers in August. Grows 2 feet high, on a dry hill three miles north-west of Cincinnati. Specimens from Mr. Lea.

L. sessiliflora, *Nutt*. Purple flowers,—appearing in August. Grows 2 or 3 feet high. Dry hill, four miles north of Cinnati. *T. G. Lea, Esq.*

L. procumbens, *Michx*. Hill-side, Bank lick, Ky. About Cincinnati. *T. G. Lea, Esq*. Meadville, Penn. *Buchanan.*

Lupinus perennis, *Linn*. Wild lupine. Barrens, Dover, Tuscarawas co.

Petalostemon violaceum, *Willd*. Grows a foot high, and is said to flower in July. Prairie, near Middletown, Ohio. Found in August, when past flowering, and called, in the Synopsis, Hudsonia? ericoides?.

Lathyrus palustris, *Linn*. Marsh pea. Perennial, flowers white and purple—in July. Near Worthington, *Paddock*. Wet prairie, two miles south of Columbus. Webbsport.

L. venosus, *Muhl*. Two miles west of Cincinnati. (?)

Vicia caroliniana, *Walt*. One foot in height. Pine knob, near Boston, Western Reserve.

Psoralea latifolia, *Torr*. Has somewhat the habit of the genus Hedysarum. Flowers blue—in July. Grows in hedges, on the borders of prairies, and banks of streams, from 2 to 4 feet high. Franklin co. *Lapham*. Abundant in central Ohio, the Miami country, etc.

*Betulineæ.*

Betula excelsa, *Ait.* Yellow birch. A tree 70 or 80 feet high, flowering in May and June,—found in low grounds. *Beck.* On the margin of forest-girt swamps, Akron, where it seldom exceeds 40 feet in height.

B. lenta, *Linn.* Black birch. Large tree, found in shady, rocky situations, in the Western Reserve.

Betula pumila, *Linn.* Young branchlets and leaves silky pubescent; older branches brown, glabrous, sparingly punctate, not fragrant; *leaves* orbicular-obovate, an inch in diameter; older ones wholly glabrous, crenate, on short petioles; younger ones covered on both sides with shining pubescence, serrate, subsessile: *fertile ament*, oblong and cylindrical, 8 lines long; *scale* sinuately 3-lobed, unguilicate; *lobes* rounded, two lateral ones eared, sinuses between the lobes extending half way down; *seeds* orbicular, margin almost equalling in width the diameter of the nucleus; styles persistent. I met with this shrub on the 12th of last August, in a boggy tamerack swamp, Copley, Portage co. It rises in height from 5 to 10 feet. Having never seen authentic specimens of Betula pumila, I have thought it prudent to insert this description, especially as the B. pumila is said to grow 2 or 3 feet high.

Alnus serrulata, *Linn.* Alder. By the side of streams, etc. Licking Narrows. Abundant in the Western Reserve.

*Salicinæ.*

Salix discolor, *Willd.* Bog willow. A shrub flowering in April. Worthington. *Paddock.*

S. houstoniana, *Pursh.* Worthington. *Paddock.*

*Euphorbiaceæ.*

Phyllanthus caroliniensis, *Walt.* A slender annual, flowering in July and August, and growing on the sandy banks of streams, to the height of 12 inches. Cincinnati. Covington, Ky.

*Sapindaceæ.*

Cardiospermum halcacabum, *Willd.* Heart seed. Balloon pea. A delicate vine with small white flowers. Fence corners etc., 4 miles north of Cincinnati. *T. G. Lea, Esq.* Woods, Pickaway co. Possibly not a native.

*Acerineæ.*

Acer spicatum, *Linn.* Mountain maple. A shrub 8 to 10 feet high, flowers greenish — in May. Rocky situations, Bedford. Little Mountain.

*Anacardiaceæ.*

Rhus copallina, *Linn.* Mountain sumach. Three to ten feet high. Knobs, Kentucky, opposite Portsmouth. Licking Narrows, O. Near Cleaveland.

R. typhina, *Linn.* Stag's horn. Giant sumach. Ten to twenty feet high. Western Reserve. Knobs, Kentucky, (Licking River, Ky., *T. G. Lea, Esq.*)

*Violaceæ.*

Viola congener, *Le Conte.* The purple flowers of this stemless, perennial violet, may be seen as early as April. Chilicothe. *Ward.*

*Cistineæ.*

Lechea villosa, *Ell.* Pin-weed. Perennial, 1 to 2 feet high, flowers brown — in July. Barrens, Dover on the Ohio canal.

Helianthemum corymbosum, *Michx.* (?) *Bracts* 2, arising apparently from the calyx, linear or acerose, shorter than the calyx, near one-half its length; *sepals* 3, valve-like, overlapping on the right hand, ovate, prominently convex, acutish, inclosing the mature seed; *capsule* roundish triangular, breadth equalling length, acutish at both ends, 3-valved, 1-celled, with spurious dissepments marking the axis of each valve: *seeds* 9,? flattened spheroidal, attached by filaments to 3 pedicles arising from the base of the valves. Whole plant covered with a dense, very short, stellular pubescence; *leaves* oblong, oval and lanceolate, entire, acutish, on short petioles, rather smaller than the leaves of Lechea villosa, crowded and somewhat appressed; *flowers* numerous, much smaller than those of H. canadense, (judging from specimens without petals,) subsessile upon the branchlets, paniculate, (not corymbed). Perennial. Stem about a foot high, assurgent and erect. Barrens, Dover, on the Ohio canal.

*Sarraceniecæ.*

Sarracenia purpurea, *Linn.* Side-saddle flower. Abundant on the cranberry marshes and quagmires of the Western Reserve. Massillon.

*Droseraceæ.*

Drosera rotundifolia, *Linn.* Sundew. Bogs, Akron.

*Lineæ.*

Linum sulcatum, *mihi.* Root annual, branching, sending up one or two sulcate stems; bark strong, like that of the cultivated flax; *leaves* sessile, lance-linear, acute, margin scabrous, midrib and margin prominent on both sides; *flowers* numerous, on erect fastigiate branchlets; *sepals* 5, lance-ovate, attenuate at the point, base setosely pectinate or ciliate, persistent; *petals* 5, yellow, obovate, one-third longer than the sepals; *stamina* 5; *styles* 5; capsule globose, 10-valved, 10-celled, each cell containing an oval, compressed, oily seed. Stems 2 feet high, leaves mostly three-fourths of an inch long, and one line wide, upper leaves minute. Grows on dry barrens, Dover, Tuscarawas co. Flowers in August.

*Caryophylleæ.*

Silene antirrhina, *Linn.* Sleepy catch-fly. Grows 1 or 2 feet high, has small, obscure, whitish flowers—in June. Sandstone Knobs, Ky., opposite Portsmouth. Near Chilicothe.

S. nivea, *Muhl.* Flowers white, developed in July. Growing near 2 feet high, on Mill Creek bottom, Cincinnati. A single plant was found last summer, by Mr. Lea.

S. nocturna, *Linn.* Flowers white, flowering in July, annual. Dayton. *M. G. Williams.*

Cerastium hirsutum, *Muhl.* Hairy chickweed. Columbus. *Lapham.*

*Amarantaceæ.*

Amarantus spinosus, *Linn.* Grows 16 to 18 inches high, flowers in July. Banks of the Ohio river, Cincinnati. *Lea.* Introduced.

*Chenopodeæ.*

Chenopodium hybridum, *Linn.* Grows from 2 to 3 feet high, on road-sides, etc. It is an annual, and is said to have been introduced. Dover.

Blitum capitatum, *Linn.* Strawberry blite. Indian strawberry. This curious little annual herb, grows about 15 inches high, in fields and by-places. It flowers in June. Akron.

*Polygoneæ.*

Polygonum fluitans, *Eat.* Stems 10 to 20 feet long, with myriads of swimming rootlets. Corresponds perfectly with Eaton's description, Manual, 6th ed., p. 274. Summit Lake, near Akron.

*Ericeæ.*

Andromeda caylculata, *Linn.* Three to four feet high. Bogs, Akron. Cleveland.

A. racemosa, *Michx.* Marsh. Marietta.

*Elæagneæ.*

Shepherdia canadensis, *Nutt.* Sea buck-thorn. Shrub 6 to 8 feet high, growing in a sand-stone ravine, Bedford, Western Reserve.

*Vaccineæ.*

Vaccinium corymbosum *Linn.* var. ——? Fruit black, of a mawkish sweet taste, one-third smaller than that of V. corymbosum; branchlets cinereous, not green, 3 to 4 feet high, being much smaller than the V. corymbosum with which it grows. In bogs, Copley swamp, Portage co., O. Under surface of the leaves punctate with resinous spots, venation remarkably translucent.

*Pyrolaceæ.*

Pyrola secunda, *Linn.* Flowers greenish-white, secund; stem 2 to 3 inches high. Little Mountain. Bedford.

Chimaphila umbellata, *Pursh.* Pipsissawa. Western Reserve. Little Mountain.

*Compositæ.*

Carduus pumilus, *Nutt.* This species exceeds all other native thistles in the size of its flower, at the same time it is remarkably low, varying from 1 to 2 feet in height. Flowers purple,—in August, root biennial. Barrens about Dover.

C. repandus, *Michx.* Perennial, 2 feet high, in flower in June. In the specimens which I have met with, the flowers are generally in threes, and the leaves are not very "closely fringed with spines." (Ell. ii. 269.) Dry sandstone knob, Ky., opposite Portsmouth. Hills south-west of Chilicothe.

C. muticus, *Nutt.* Wet prairie on Mad river, eight miles east of Dayton.

Gnaphalium purpureum, *Linn.* Dry, woodland hills, 3 miles north of Cincinnati. *T. G. Lea, Esq.*

Aster novi-belgii, *Linn.* Columbus, *Lapham.* Dayton, *Van Cleve.*

A. polyphyllus, *Willd.* Prairie, Dayton. Specimen from *M. G. Williams.* Much branched, perennial, two feet high, flowers in September. Abundant in meadows and fields, New Albany, Ia. *Dr. Clapp.*

Eupatorium rupestre, *Raf.* (Where described?) I found a plant in the Miami country, with pale yellow flowers, 2 to 3 feet high, perennial, to which Dr. Short gives the above name.

Cacalia tuberosa, *Nutt.*? Agrees with Nuttall's description, (Genera, ii, 138,) with these exceptions:—*Lower leaves* spatulate and oval, instead of ovate; *upper leaves* with many prominent lateral teeth, not entire; about three feet high, instead of 4 to 6 feet. Corymb many flowered, scales of the involucre carinate, 5 to each flower, embracing 5 florets, leaves 5 or 7-nerved. The character of the root, I do not know. It may prove a species heretofore undescribed. Wet prairie, 2 miles south of Columbus. Flowers white,—in July.

Helianthus occidentalis, *mihi.* I give this specific name provisionally, being uncertain whether the plant may not have been vaguely described already. *Root* perennial, small, fusiform and fibrous; *stem* simple, pilose; *petioles* very hairy; *leaves* opposite, mostly radical, and on long footstalks, lance-ovate, acutish, attenuated into the peotiles, remotely dentate-serrate, ciliate, 3-nerved, hispid and scabrous on both sides; *upper leaves* very small, entire, subsessile, on short ciliate petioles; *flowers*, in my specimens, single or in threes, naked; *peduncles* hispid; *scales of the involucre* broad-ovate, cuspidate, densely ciliate, equalling the disc; *rays* 12 to 14; *disc* yellow; *chaff of the receptacle* tricuspidate, middle division much the largest; *pappus* membranaceous, fringed with two long pointed projections. Stem 2 to 3 feet high, lower leaves 1 inch broad and 4 inches long, on petioles of equal length. Dry barrens, Dover, Tuscarawas co. Flowers in August, flowers yellow, an inch in diameter.

Coreopsis verticillata, *Willd.* Grows on the rocky heights, across the river from Portsmouth. Flowers yellow,—in June, 2 to 3 feet high. The leaves are lanceolate like those of C. senifolia, and mostly glabrous like those of C. verticillata. This may be the C. senifolia, *Michx.* I cannot perceive any essential differences in Elliott's descriptions of the two species, at least, in those parts which the immaturity of my specimens permit me to inspect. Mrs. Dupuy says this plant is often procured from the Alleghany glades, and after being wilted in an oven, is used as a substitute for tea.

Senecio aureus, *Linn.* Rag-wort. False valerian. Columbus. *Lapham.*

Polymnia uvedalia, *Linn.* Bearsfoot. Woody hill-sides, a few miles north of Roscoe.

*Stellatæ.*

Galium lanceolatum, *Torr.* Sandstone knobs and hill-sides, Chilicothe. Kentucky, opposite Portsmouth.

G. trifidum, *Linn.* Small cleavers. Wet meadows, marshes, Cincinnati, *Lea.* Webbsport.

*Caprifoliaceæ.*

Lonicera ciliata, *Muhl.* Fly honey-suckle. Twin berry. A shrub, from 2 to 4 feet high. Flowers yellow, appearing in May. Rocky, hilly situations. Bedford, Western Reserve.

Cornus canadensis, *Linn.* Low cornel. Dry saw mill, Newark. Western Reserve.

Viburnum dentatum, *Linn.* Arrow-wood. Eight feet high. Swampy woods, Western Reserve.

Diervilla canadensis, *Muhl.* Bush honey-suckle. Ohio generally.

*Asclepiadeæ.*

Acerates longifolia, *Michx.* *Eu.* i. 317. 18 to 24 i. 4. Wet prairie. Scott's plains, Franklin co.

A. viridiflora, *Raf.* Leaves oblong, ocute. Two feet. Dry prairie, Middletown, Miami country.

*Gentianeæ.*

Menyanthes trifoliata, *Linn.* Buck-bean. Marshy woods, Akron. Cleveland.

Gentiana rubricaulis, *Schw.* One foot high. Dayton. Rare. *M. G. Williams.*

*Polemoniaceæ.*

Phlox subulata, *Linn.* Mountain pink. Three to four inches high. Hills, Newark.

*Labiatæ.*

Lycopus obtusifolius, *Vahl.* Prairies, central Ohio. *Paddock.*

Scutellaria galericulata, *Linn.* Marshes, Webbsport. Walnut Plains.

S. saxatilis, *mihi.* *Roots* filiform, creeping and stoloniferous;

*stems* always seemingly erect, but really assurgent, square, pilose, sending off branches near the root, simple above; *leaves* pilose on both sides, ciliate, rugose; lower ones round, cordate, laterally and remotely crenate, entire near the base and apex, obtuse, from 2 lines to an inch in diameter, on pilose petioles of greater length; upper leaves lance-ovate, obtuse, with two or three lateral crenatures, abruptly attenuated into short petioles, or sessile; *bracts* lance-oval, obtuse, densely pilose and ciliate; *flowers* in a simple terminal raceme, in pairs, at intervals of nearly half an inch; *calyx* hispid; *corol* pubescent without, glabrous within, three-fourths of an inch long, deep blue, excepting the under side of the tube, which is white; *anthers* pubescent; *filaments* dilated and pilose in the middle. From 6 to 12 inches high. Growing on the arid cliffs, Ky., opposite the mouth of the Scioto. Dry hill, 3 miles south-west from Chilicothe. Flowers in June. The S. pilosa, to which I at first hesitatingly referred it, is described as having "the lower leaves slightly cordate; racemes paniculate, with the flowers crowded; upper leaves nearly acute." (Ell. ii. 90.) "Leaves attenuated into the petiole, flowers whitish, in paniculate racemes." (Beck, 282.) The disagreements do not require pointing out. I have a Scutellaria, procured by Dr. Paddock, near Morgantown, Va., certainly distinct from this, and which agrees very well with the descriptions of S. pilosa.

Stachys cordata, *mihi.* Perennial, whole plant lanuginose, being clad with white, terete, pointed hairs, the larger ones always jointed, the smaller, often crowned with glands or stellular branches; *stem* simple, erect, with four prominent rounded corners, and as many rounded intervening grooves, hairs upon the stem horizontal; *leaves* cordate, oval or ovate in outline, acute, regularly crenate and ciliate, two inches long by one and a half in width, lower leaves on petioles of one-third their own length, those immediately below the spike of flowers, ovate, subcordate, crenate-serrate, on very short petioles; *bracts* oblong, entire; *calyx* with 5 acute, subspinous teeth; *corol* mottled, red and white, middle lobe of the lower lip rounded; *flowers* sessile, in whorls of six, at intervals generally of half an inch. Grows in woods, to the height of 18 inches or 2 feet, throughout the middle, southern, and western portions of Ohio. Flowers from June to August. Perhaps it is the S. syvlatica of Nuttall, which Beck describes as having "acuminate, serrate leaves; floral leaves nearly linear." (Beck, 279.) Eaton says, "the stem of S. sylvatica is "prickly backwards." In the Synopsis of Western Plants, I introduced the species here described, as S. intermedia, *Ait.* (?); but that species is said to have many-flowered whorls, and oblong subcordate leaves. I have inspected a great number of living plants, as well as many dried specimens, and have uniformly found them characterized as I have described.

S. glabra, *mihi.* Perennial, generally glabrous; *root* descending, sub-fusiform, branching; *stem* branching, with 4 prominent corners and intervening shallow grooves; a few straggling hairs and reversed prickles near the root; *branches* divaricate; *leaves* oblong, seldom oval, acute; lower ones 2 inches long by 1 in width, truncate at the base, on petioles half an inch long; upper ones attenuated into the petioles, subsessile; *bracts* lance-ovate, longer than the flowers, with two lateral serratures; *flowers* generally whorled in sixes, at intervals of half an inch; *calyx* with 5 equal, lance-subulate, subspinous teeth; *corol* variegated with red and white, corresponding to the generic description of Stachys. About a foot high. Flowers in August. Grows on the banks of streams, and in low woods. Miami country. Worthington. Roscoe.

*Boragineæ.*

Echinospermum lappula, *Lehm.* This noisome weed has the odor of rats. It grows by roads sides, Chillicothe. Newark. Akron. It is annual, has blue flowers, and grows a foot high.

*Coniferæ.*

Juniperus communis, *Linn.* Juniper. Meadville Penn. *R. Buchanan.* Bedford O.

Taxus canadensis, *Willd.* Dwarf yew. Bedford. Little Mountain.

*Alismaceæ.*

Sagittaria rigida, *Pursh.* Flowers in July, flowers white. Grows in standing pools of water. Rather variable as a species. Newark, Licking county.

*Xyrideæ.*

Xyris brevifolia, *Michx.* A foot high, flowers yellow, leaves short, grass-like. Flowers in July and August. Found growing in peat earth, margin of the Summit lake, Akron.

*Orchideæ.*

Spiranthes gracilis, *Beck.* ? Hills, 4 miles north of Cincinnati. *Lea.* 4 to 10 inches high.

Goodyera repens, *Brown.* Root creeping, stem 6 to 8 inches high, flowers greenish white. Little Mountain.

Orchis rotundifolia, *Pursh.* Worthington. *Paddock.*

Habenaria tridentata, *Hook.* Shady swamp, Copley, Portage county.

H. herbiola, *Brown.* Grows in swamps, from 12 to 18 inches high, perennial, flowers small and greenish,—June. Worthington. *Paddock.*

H. blephariglottis, *Hook.* A fine, white-flowered species, growing in bogs, among the Sphagnums and cranberries, Akron. Perennial, 18 to 24 inches high.

H. ciliaris, *Brown.* Damp woods, Cleveland.

H. psycoides, *Spreng.* A perennial, pale yellow flowered species, growing two feet high, in damp woods, Copley swamp, Portage co. Flowers in July and August.

H. retusa, *mihi.* Lip with a prominent, obtuse palate, three-lobed, the intermediate lobe retuse, 4 times larger than the lateral ones, which are rounded and separated from it by repand sinuses; outer segments of the perianth oval, inner ones orbicular; *spur* clavate, obtuse, equalling the germ; *bracts* lanceolate, alternately acute, shorter than the germ; *leaves* on the lower part of the stem lance-oblong, acute; *flowers* greenish yellow, equalling in size those of Goodyera repens, appearing in August. I have met with a single individual plant, answering to the above description. Barrens a mile east of Dover, Tuscarawas county.

Cypripedium acaule, *Ait.* Woods, Western Reserve.

*Junceæ.*

Luzula pilosa, *Willd.* Hairy wood-rush. Perennial, 6 to 12inches high, hairy, growing in dry woods, and flowering in April and May. Oxford. Portsmouth. Newport, Kentucky.

*Melanthaceæ.*

Veratrum angustifolium, *Pursh.* Grows in a wet meadow half a mile south of Massillon, to the height of 2 to 4 feet. Darby plains.

*Pontedereæ.*

Pontederia cordata, *Linn.* Pickerel weed. Ponds, etc., Akron.

*Smilaceæ.*

Streptopus lanuginosus, *Michx.* Western Reserve.

*Aroideæ.*

Calla palustris, *Linn.* Water arum. Procumbent in swamps, ascends 6 to 8 inches, flowers white and green. Western Reserve.

*Fluviales.*

Potamogeton perfoliatum, *Linn.* Perfoliate pond-weed. Flowers greenish yellow, stems 12 to 18 inches high. Canal 5 miles north of Roscoe.

P. lucens, *Linn.* Shining pond-weed. In the Summit lake, and canal, near Akron.

P. zosterifolium, *Trin.* Flowers greenish yellow,—in July. Stems 2 to 3 feet long, in a pool of clear water, Licking narrows.

*Gramineæ.*

Poa capillaris, *Linn.* Miami country. *Frank**.

P. angustifolia, *Linn.* ? One or two feet high, flowers in June. Cincinnati. *Frank.*

Polypogon racemosum, *Nutt.* Beard grass. Cincinnati. *Buchanan.*

Trichodium scabrum, *Muhl.* Culm 18 inches high. Cincinnati. *Buchanan.*

T. elatum, *Pursh.* Culm 2 feet high, flowers in September. New-Albany, Ia. *Clapp.* Cincinnati. *Buchanan.*

Trisetum subspicatum, *Brown.* Perennial, 12 to 18 inches high, flowers in June. Dupuy's knob, Greenup co., Ky. Dry hill, Chillicothe.

Bromus mollis, *Linn.* Biennial, 3 feet high, flowers in July. Barrens, Dover, Tuscarawas co.

B. pubescens, *Muhl.* Bigelow's marsh, Cincinnati. *Frank.*

Festuca nutans, *Willd.* Wet meadows, Cincinnati. *Frank.*

*Cyperaceæ.*

Eriophorum polystachyon, *Linn.* Swamps, Western Reserve.

E. virginicum, *Linn.* Virginian cotton grass. Perennial, 2 to 4 feet high, flowers in August. Swamps, Akron.

Carex setacea, *Dew.* Ditches, Cincinnati. Flowers in June. *Frank.*

C. lupulina, *Muhl.* Wet meadow near the Four mile House, Cincinnati. *Frank.*

C. rosea, *Schk.* Moist woods, flowers in May. Columbus. *Lapham.*

C. laxiflora, *Lam.* Culm 1 to 2 feet high, flowers in April. Woods. Scarce. Cincinnati. *Frank.*

---

*Dr. Jos. C. Frank, a most zealous and accomplished botanist, author of "Rastadts Flora," (Heidleberg, 1830,) was deputed by some German society to travel in this country and make botanical collections. He spent a year in Cincinnati before setting out, during which time he gave special attention to our grasses and carices. Unfortunately he fell a victim to the yellow fever, by exposing himself too early, last autumn, to the climate of New Orleans. Dr. Frank left me specimens of all the species introduced into this catalogue on his authority.

C. crinita, *Lam.* Flowers in June, culm 2 to 4 feet high. Marshes, Cincinnati. *Frank.*

C. aristata, *Brown.* Culm 12 to 18 inches high. Shady woods, Miami country. ? *Frank.*

C. cristata, *Torr.* Culm 2 feet high, flowers in June. Shady woods, Cincinnati. *Frank.*

C. virescens, *Muhl.* Culm 12 to 18 inches high, growing in open woods, Miami country. ? *Frank.*

C. oligocarpa, *Schk.* Culms 4 to 6 inches high, flowers in May. Open woods, Germantown. *Frank.*

C. multiflora, *Muhl.* Culm 2 feet high, flowers in June. Wet meadows, Cincinnati. *Frank.*

C. conoidea, *Schk.* Culm a foot high, flowering in June. Shady woods and wet meadows, Cincinnati. *Frank.*

C. pubescens, *Muhl.* Miami country. *Frank.*

C. granularis, *Muhl.* Cincinnati. Germantown. *Frank.*

C. arida, *Torr.* Dry pastures, Cincinnati. Germantown. *Frank.*

C. gracillima, *Schw.* Culm 18 to 24 inches high, flowers in May and June. Grows in open woods, Germantown. *Frank.*

*Filices.*

Polypodium phlegopteris, *Linn.* Pale mountain polypod. On a steep, shaded, sandstone hill-side. Dry saw-mill, Newark.

P. dryopteris, *Linn.* A most delicate triangular fern, perennial, root creeping, black, small; stipe 6 to 10 inches high; in fruit in July. With the last, Newark.

Woodwardia virginica, *Willd.* (?) The stipe of this tall, rank fern, is dark below; it grows plentifully in the tamerack swamps of the Western Reserve, to the height of 3 to 5 feet. In fruit in July and August. Accords with Beck's description (Bot. 454) in all but the following particulars: Pinnæ lance-linear, instead of lanceolate; segments obtuse, instead of acute; stipe chaffy near the root, instead of smooth. Beck says the W. virginica is 2 feet high; Eaton says 1.

Aspidium dilatatum, *Willd.* A perennial fern, 2 feet high, in fruit in July, growing in shady woods, Western Reserve. Webbsport.

A. noveboracense, *Willd.* Perennial, frond 18 inches high, fruit or sporules mature in July and August. Gregarious, growing in damp woods, Newark and Webbsport. Compares with a specimen taken by Dr. Torrey, from the vicinity of New York.

A. angustum, *Willd.* Perennial, mature in July, growing in meadows etc., to the height of 18 inches. Meadville. *Buchanan.* Massillon. Cincinnati.

A. filix-fæmina, *Willd.* Perennial, frond 18 to 24 inches high, growing in low shady grounds. Specimens from Meadville, procured by Mr. Buchanan; from the neighborhood of Cincinnati, by Mr. Lea.

A. spinulosum, *Willd.* (?) Having entertained some doubt in respect to this species, at the time of publishing the Synopsis of Western Plants, and having never met with it except in the low, damp forests of central and north-eastern Ohio, I am induced to add the following description: *Stipe* and *rachis* chaffy, the latter rather longer of the two; *frond* bipinnate, general outline lance-oblong, glabrous; *pinnæ* sub-opposite, from 12 to 20 pairs; lower ones triangular from the prolongation of their lower pinnules, as large, though not as long as the middle pinnæ; upper ones lanceolate; *pinnules* lance-oblong, decurrent, often deeply gash-pinnatifid; *segments* often oblong and oval, with from 3 to 7 appressed, mucronate serratures; *sori* round, large, (intermediate between those of A. dilatatum and of A. marginale,) distinct, equidistant from the midrib and margin, corresponding to the segments or their serratures, and seldom if ever occurring on the lower pinnæ. Frond generally 16 inehes long, and 5 or 6 in width, of a dark green color. Grows in damp, shady woods, in colonies of four or five, to the height of more than 2 feet.

A. intermedium, *Willd.* (?) The specimens which I have hesitatingly referred to this species, were found near Marietta, and by Mr. Buchanan near Meadville, Penn. *Fern*

2 feet high, *stipe* somewhat chaffy, *frond* lanceolate, almost lance-linear, hardly bipinnate; *pinnæ* lanceolate, deeply pinnatifid, *pinnules*, if they may be so called, linear-oblong and obtuse in outline, serrate with every second or third gash deeper, the pinnules next the rachis, on the lower pinnæ, are nearly or quite distinct, thus rendering the frond to the same extent bipinnate; *sori* oval, large, arranged in rows on each side of the midrib of the pinnule, becoming confluent; *indusium* opening inwards. This species differs from what I have taken to be the A. dilatatum, in having the sori oval instead of round, in being circumscribed by a much narrower outline, and in being hardly bipinnate, while the latter is often clearly tripinnate. Moreover, the A. dilatatum has mucronate serratures, which this species has not. The three admitted species, A. filix-fæmina, A. asplenoides and A. angustum, much more closely resemble each other, than do these two.

Dicksonia pilosiuscula, *Willd.* Frond 2 to 3 feet high, yellowish green, sori mature in July and August, gregarious. Grows in arid, argillaceous soils. Columbus. *Lapham.* Slaty ravine, 2 miles north of Worthington. Habit much like that of Aspidium noveboracense.

Hypopeltis obtusa, *Willd.* Rocky situations, Roscoe. Chillicothe.

Osmunda claytoniana, *Linn.* Resembling osmunda cinnamonea, *Linn.* One to two feet high, in fruit in August; grows in marshy woods, Copley swamp, near Akron.

*Lycopodiaceæ.*

Lycopodium complanatum, *Linn.* Newark. Cleveland.

L. dendroideum, *Michx.* Ground pine. Damp woods, Cleveland.

L. lucidulum, *Michx.* Creeping, branches 3 to 5 inches high, perennial, grows in slaty ravines, Franklin co. *Lapham.* Western Reserve. L. annotinum has not been observed in Ohio.

*Musci.*

Sphagnum latifolium, *Brid.* Peat moss. Color pale green, yellow and reddish, stem 6 to 12 inches long; spread like a bed or carpet over the shaking bogs, Western Reserve.

Splachnum setaceum, *Brid.* ? Pale green. Similar to the last in habit, size, and location, though preferring the shaded to the open bogs. Akron.

*Hepaticeæ.*

Riccia fluitans, *Linn.* Fork stems. Floating on water, dichotomously branched, green, having some resemblance to Lemna trisulca. Shaded pool 2 miles north of Newark, Knox co. Found in July.

---

### Catalogue of Plants lately found in the States bordering on Ohio.

Those species published by Dr. Short, in the Transylvania Journal, as having been discovered by himself and Mr. Griswold in Kentucky, are not included. This list, as will be seen by inspection, has been greatly augmented by the contributions of A. Clapp, M. D. of New-Albany, Ia., on whose accuracy reliance can be placed.

Cnidium canadensis, *Spreng.* (?) *Stem* 12 to 18 inches high, flexuous; *leaves* pinnate, 6 or 8 pairs, leafets gash-pinnatifid, or gash-toothed, lance-ovate; *general involucre* 4 or 5 leaved, linear, sometimes trifid; partial one 6 to 8 leaved, linear, ovate; *petals* white, nearly obcordate; *rays* of the umbels and umbellets corresponding in number generally, but vaguely, with the leaves of the general and partial involucres. Growing in water, Bronson, Michigan. Specimen from Mr. Williams of Buffalo.

*Ranunculaceæ.*

Ranunculus prostratus, *Eat.* R. clintonii, *Beck.* Not rooting at the joints. On rich grounds periodically inundated, New-Albany, Ia. *Clapp.*

*Hypericineæ.*

Hypericum petiolatum, *Walt.* Perennial, flowers dull red,—in August, stem 2 feet high, grows in wet places, New-Albany. *Clapp.*

*Rosaceæ.*

Potentilla arguata, *Pursh.* Flowers pale yellow. Indiana. Specimen from Mr. Dunbar of Indianapolis.

*Pomaceæ.*

Aronia latifolia, *mihi.* *Leaves* broad oval, nearly orbicular, cordate and subcordate at base, (excepting those at the extremities of the branches,) dentate-serrate, entire at base, abruptly acute or sub-mucronate at the apex, about two and a half inches long and two inches wide, upper surface glabrous, under surface near the base tomentose; *petioles* an inch long, tomentose; *racemes* few-flowered; *calyx* superior; *sepals* 5, ovate cuspidate, tomentose; *petals* lance-linear, ? obtusish, ? much longer than the sepals: *stamens* 20, ? arising from the calyx; *styles* 5; *pome* globose, 5-celled, each cell 2-seeded, some of them abortive; *seeds* smooth, oval, compressed, hooked at base. A shrub 6 or 8 feet high, with brown, punctate branches, growing among sandstone rocks, near the summit of Dupuy's knob, Ky., a mile south of Portsmouth, O. Observed in June last, when the immature fruit was as large as peas, and the crisped remnants of petals were rarely to be found. This may prove synonymous with Pyrus ovalis, *Linn.*, or P. sanguainea, *Pursh.* I propose a temporary name, accompanied with the above description, with a view of eliciting truth.

*Leguminosæ.*

Hedysarum canescens, *Linn.* H. scaberrimum, *Ell.* Four feet high, flowers in August. North shore of the Falls of the Ohio. *Clapp.*

Amorpha canescens, *Nutt.* Lead plant. Bronson, Michigan. Specimens from Mr. Williams, of Buffalo. Indianopolis. Specimens from Mr. Dunbar.

*Cupuliferæ.*

Quercus triloba, *Linn.* Downy black oak. Twenty feet high. Knobs, Greenup co., Ky., with Pinus variabilis.

Q. rubra, *Linn.* Red oak. Large tree, quite frequent in moist, rich, rather wet soils, New Albany, Ia. *Clapp.*

Q. catesbæi, *Michx.* Barren scrub oak. A shrub, growing on the knobs and barrens back of New Albany, Ia. *Clapp.*

*Betulineæ.*

Alnus glauca, *Michx.* Black alder. Meadville, Penn. *Buchanan.*

*Salicineæ.*

Salix petiolaris, *Willd.* A small tree, 6 or 8 feet high, flowering in April, growing at the head of Sandy Island, near New Albany. *Clapp.*

*Geraniaceæ.*

Geranium robertianum, *Linn.* Herb robert. Annual, 10 or 12 inches high, flowers small, purple, appearing from June to October. Plant fetid, growing in rocky shaded places, Meadville, Penn. *Buchanan.*

*Polygaleæ.*

Polygala cruciata, *Nutt.* Annual, flowering from July to September, flowers greenish purple. Six to twelve inches high, growing in wet places, Bronson, Michigan. Specimens from Mr. Williams.

*Violaceæ.*

Viola hastata, *Michx.* Ten to twelve inches high, flowers yellow, appearing in May. Morgantown, Va. *Paddock.*

*Campanulaceæ.*

Campanula rotundifolia, *Linn.* Flax bell-flower. Perennial, a foot high, flowers blue,—in July, grows in rocky situations, Bronson, Michigan. *Williams.*

*Compositæ.*

Sonchus carolinianus, *Walt.* Annual, flowers yellow,—in

August, 2 feet high, growing in fence corners in fertile soil, New-Albany. *Clapp.*

Lactuca sagittifolia, *Ell.* Growing in fields of second-rate land, New-Albany. *Clapp.*

L. sanguinea, *Bigelow.* Flowers rather later than L. elongata, grows in fields, rare, New-Albany. *Clapp.*

Aster patens, *Ait.* (Beck, 183.) Perennial, 18 inches high, flowers in July and August. Dry sandstone knobs, New-Albany. *Clapp.*

A. sagittifolius, *Willd.* Two feet high, flowers blue,—in September and October, rare, growing on the banks of streams. New-Albany. *Clapp.*

A. rigidus, *Willd.* Perennial, 10 to 18 inches high. A well-marked species, growing in dry barrens, New-Albany. *Clapp.*

Solidago gigantea, *Ait.* Flowers in August, grows 5 feet high, in moderately fertile soils on high grounds, New-Albany. *Clapp.*

S. glomerata, *Michx.* Flowers in September, grows 18 inches high, on dry, sterile knobs, New Albany. *Clapp.*

S. altissima, *Linn.* A robust species, growing 5 feet high, in a meadow near New-Albany. *Clapp.*

S. rupestris, *Rafinesque.* (Where described?) Flowers in September, 16 to 18 inches high. Rocky situations, north bank of the Falls of the Ohio. *Clapp.*

Eupatorium trifoliatum, *Linn.* Flowers white,—in September, growing in woods among the knobs, New-Albany. *Clapp.* Western states. *Beck.*

E. serotinum, *Michx.* Flowers white,—in September and October, 3 to 4 feet high, growing on the banks of sandy rivulets, New-Albany. *Clapp.*

Helianthus divaricatus, *Linn.* Grows 5 or 6 feet high, in fence corners, etc., New-Albany. *Clapp.*

*Stellatæ.*

Galium boreale, *Pursh.* Bronson, Michigan. *Williams.*

*Polemoniaceæ.*

Phlox acuminata, *Pursh.* Flowers purple,—in August. Grows 3 to 5 feet high, in fertile soils, New-Albany. *Clapp.*

*Solaneæ.*

Solanum virginianum, *Linn.* Flowers bluish-white, stem 18 inches high, growing on river banks, New-Albany. Rare. *Clapp.*

*Labiatæ.*

Salvia urticifolia, *Linn.* Nettle-leaf sage. Growing among knobs, Ia., 5 or 6 inches high. Rare. *Clapp.*

Pycnanthemum monardellum, *Michx.* Perennial, growing from 2 to 3 feet high, flowers whitish-red,—in July. Morgantown, Va. *Paddock.*

Stachys hyssopifolia, *Michx.* Perennial, stem 8 to 12 inches high, flowering in July and August. Borders of wet meadows, Prairie Ronde, Michigan. Specimens from Mr. Williams.

*Boragineæ.*

Lycopsis virginica, *Linn.* (?) Found with mature seed, about the middle of June, on an arid knob, Greenup co., Ky. Annual, whole plant hispid; *stem* erect, 6 inches high, branching; *leaves* lance-oval, obtuse, half an inch long and two inches wide, sessile, lower ones obovate; *flowers* in long terminal racemes; *pedicels* as long as the calyx; *teeth* of the calyx lanceolate, acute, not closing; *seeds* ovate, lenticular, margined, smooth and shining.

Lithospermum officinale, *Linn.* Common gromwell. One to two feet high, flowers pale yellow, growing on argillaceous hill-sides, New-Albany. *Clapp.* Introduced.

*Orchideæ.*

Pogonia ophioglossoides, *Brown.* About Coneaught lake, Penn. *Buchanan.*

*Melanthaceæ,*

Veratrum viride, *Ait.* Poke root. American Hellebore. Neighborhood of Meadville, Penn. *Buchanan.*

Tofielda pubescens. *Pursh.* Perennial, 18 inches high, flowers in July, grows in marshes, Bronson, Michigan. *Mr. Williams*, of Buffalo.

*Smilaceæ.*

Smilax sarsaparilla, *Linn.* Stem climbing, 2 to 3 feet high, leaves glaucous, growing in barren fields, New-Albany. Rare. Not the commercial sarsaparilla. *Clapp.*

Convallaria biflora, *Walt.* Grows in moist, rich meadows, flowers in May and June, attains the height of 18 inches. New-Albany. *Clapp.*

*Gramineæ.*

Agrostis virginica, *Linn.* Culms procumbent, a foot high. New-Albany. *Clapp. Griswold.*

Panicum hispidum, *Muhl.* Culm 3 to 4 feet high. New-Albany. *Clapp.*

Avena præcox, *Linn. Eat.* Dwarf oats. An interesting little annual, 6 to 12 inches high, flowers in May and June. Grows on high, arid, sandstone knobs, with Pinus variabilis and Rhus copallina, Greenup co., Ky.

*Filices.*

Botrychium simplex, *Hitchcock.* Frond ternately bipinnatifid, gashed; spike decompound. Perennial, 6 inches high, growing in pine woods, Meadville, Penn. Specimens from R. Buchanan, Esq.

